# Theoretical Study of ClOO + NO Reaction: Mechanism and Kinetics

**DOI:** 10.3390/molecules22122121

**Published:** 2017-12-01

**Authors:** Nan-Nan Wu, Shun-Li Ou-Yang, Liang Li

**Affiliations:** 1 Key Laboratory of Integrated Exploitation of Bayan Obo Multi-Metal Resources, Inner Mongolia University of Science and Technology, Baotou 014010, China; 2 Institute of Theoretical Chemistry, State Key Laboratory of Theoretical and Computational Chemistry, Jilin University, Changchun 130023, China; 3 College of Physics, Jilin University, Changchun 130012, China; lliang@jlu.edu.cn

**Keywords:** kinetics, reaction mechanism, NO, ClOO

## Abstract

Theoretical investigations are performed on mechanism and kinetics of the reaction of halogen peroxy radical ClOO with NO radical. The electronic structure information for both of the singlet and triplet potential energy surfaces (PESs) is obtained at the MP2/6-311 + G(2df) level of theory, and the single-point energies are refined by the CCSD(T)/6-311 + G(2df) level. The rate constants for various product channels of the reaction in the pressure range of 1-7600 Torr are predicted. The main results are as follows: On the singlet surface, the addition-elimination mechanism is the most important. First, the N atom of the NO radical can attack the O atom of the ClOO radical to form an energy-riched intermediate IM1 ClOONOtp (21.3 kcal/mol) barrierlessly, then IM1 could isomerizes to IM2 ClOONOcp (22.1 kcal/mol) via a low energy barrier. Both IM1 and IM2 can dissociate to the primary product P_1_ ClNO + ^1^O_2_ and the secondary product P_2_ ClO + NO_2_. On the triplet surface, the direct Cl-abstraction reaction is the most feasible pathway. The Cl-abstraction can take place via a van der Waals complex, ^3^IM1 ONClOO (4.1 kcal/mol), then it fragments readily to give P_1_’ ClNO + ^3^O_2_ with a small barrier. The kinetic calculations show that at low temperatures, the singlet bimolecular product P_1_ is the primary product, while at high temperatures, the triplet product P_1_’ becomes the primary one; only at high pressures and low temperatures, the unimolecular products IM1 and IM2 can be found with quite small yields. At experimentally measured temperature 213 K, ClNO is the primary product in the whole pressure range, which is consistent with the previous experiment. The present study may be useful for further experimental studies for the title reaction.

## 1. Introduction

Halogen oxide has attracted wide experimental and theoretical attentions for its important role in many chemical processes, such as stratospheric ozone depletion, water disinfection, pulp bleaching, food preservation, etc. [1,2,3,4,5,6,7,8,9,10,11,12,13,14,15,16,17,18,19,20,21]. In atmosphere, halogen oxide mainly exists in free radicals, such as XO, OXO and XOO. Asymmetrical XOO is the most stable structure of halogen oxide XO_2_ [22]. The free radical (ClOO) contains a very weak chlorine-oxygen bond (Cl-O) with a bond energy of smaller than 19.5 KJ∙mol^−1^. ClOO is often described as a helium atom “accompanied with” an oxygen molecule [23]. 

In the stratosphere, there’s a reaction that will destroy the ozone:

Cl + O_3_→ClO + O_2_ (1)

This reaction is accompanied with:

Cl + O_2_ + M→ClOO + M (2)

And

ClO + ClO→ClOO + C
(3)

In these reactions, ClOO is an important intermediate and is vital to circulation of ozone depletion [23]. Therefore, elimination reactions of ClOO by other chemical elements like NO, NO_2_ and hydrocarbon in the atmosphere will influence the circulation of such chlorine-induced ozone depletion significantly.

On the other hand, NO, one of the major oxynitrides with wide distributions in atmosphere, not only is closely related with atmospheric pollutions (e.g., acid rain and photochemical smog), but also will destroy the ozone sphere [24]. Experimental and theoretical research on the generation and elimination reaction mechanism of NO has been one of important topic in atmosphere and combustion chemistry [25,26,27,28,29,30,31,32,33,34,35]. As a result, the ClOO + NO reaction has important potential significance to eliminate atmospheric pollutants and reduce ozone depletion. 

For ClOO + NO reaction, there are three feasible reaction channels in thermodynamics:

ClOO + NO→ClO + NO_2_ (4)

ClOO + NO→ClNO + O_2_ (5)

ClOO + NO + M→ClONO_2_ + M (6)

However, there has only been one experimental research project on kinetic of ClOO + NO reaction as far as we know. In 2006, Enami et al. [36] measured ClOO + NO reaction when temperature ranges from 205–243 K and pressure ranges from 50–150 Torr by using spectrographic technique under cavity environment. They discovered no obvious temperature dependence and concluded that the reaction rate remains a constant at 213 K, *k* (ClOO + NO) = (4.5 ± 0.9) × 10^−11^ cm^3^ mol^−1^ s^−1^. They speculated that the major product of ClOO + NO reaction is ClNO whose branching ratio is about 0.8. But the observed product is NO_2_ whose branching ratio is 0.18 ± 0.02 at 213 K and 0.15 ± 0.02 at 223 K. Their experimental research failed to elaborate the mechanism of this complicated ClOO + NO reaction with multiple quantum well and multiple channels, pressure and temperature dependence of its product within a wider measuring range, and product distribution. According to our knowledge, Yang et al. [37] carried out a theoretical study on this reaction at CCSD (T)/6-311 + G(2d)//B3LYP/6-311 + G(2d) level of theory in 2012. Although they have got relative detailed singlet potential energy surface, they didn’t considered triplet potential energy surface, and they didn’t calculate the kinetics of the ClOO + NO reaction. Kinetics information within other temperature and pressure range of the reaction is still not clear. In addition, recently, we have investigated the mechanism for the reaction of radical FOO with NO [38]. The calculated results show that starting from the energy-riched intermediate FOONO, FNO is the exclusive product at room temperature 298 K and at 1 Torr. Similarly, we speculate that ClNO is the primary product of the ClOO + NO reaction at experimentally measured temperature 213 K. Considering the potential significance of ClOO + NO reaction in atmospheric chemistry, it is necessary to make comprehensive theoretical studies on its mechanism and kinetic. In this paper, potential energy surface of ClOO + NO reaction was explored based on quantum chemistry calculation. A kinetic calculation was conducted by using Rice-Ramsperger-Kassel-Marcus (RRKM) unimolecular reaction rate theory of microcanonical ensemble [39], getting the reaction rate constants and branching ratios of many product channels under different temperatures and pressures.

## 2. Calculation Methods

The geometries of all of the reactants, products, intermediates, and transition states involved in the ClOO + NO reaction were optimized using the second order level of closed shell MΦller-Pleset MP2 [40] perturbation theory in conjunction with the 6-311 + G(2df) basis set. Frequency calculations were performed at the same level to check whether the obtained species is an equilibrium species (with all real frequencies) or a transition state (with one and only one imaginary frequency). To confirm that the transition states connect designated intermediates, we also performed intrinsic reaction coordinate (IRC) [41,42,43,44] calculations at the MP2/6-311 + G(2df) level. To obtain more reliable energetic data, single-point energy calculations were performed at the CCSD (T)/6-311 + G(2df) level using the MP2/6-311 + G(2df) optimized geometries of all the species. Unless noted, the CCSD(T) energies with inclusion of MP2 zero-point vibrational energies (ZPE) are used throughout. All calculations were carried out using the Gaussian 03 program packages [45]. 

According to the variational transition-state and RRKM [39] theories, the kinetic calculations for this multi-channel and multi-well reaction were carried out via the MultiWell 2011 [46,47] program on the basis of the PES obtained above in order to identify the likely mechanism and the branching ratios of various product channels.

## 3. Results and Discussion

### 3.1. Potential Energy Surface and Reaction Mechanism

**Figure 1:**
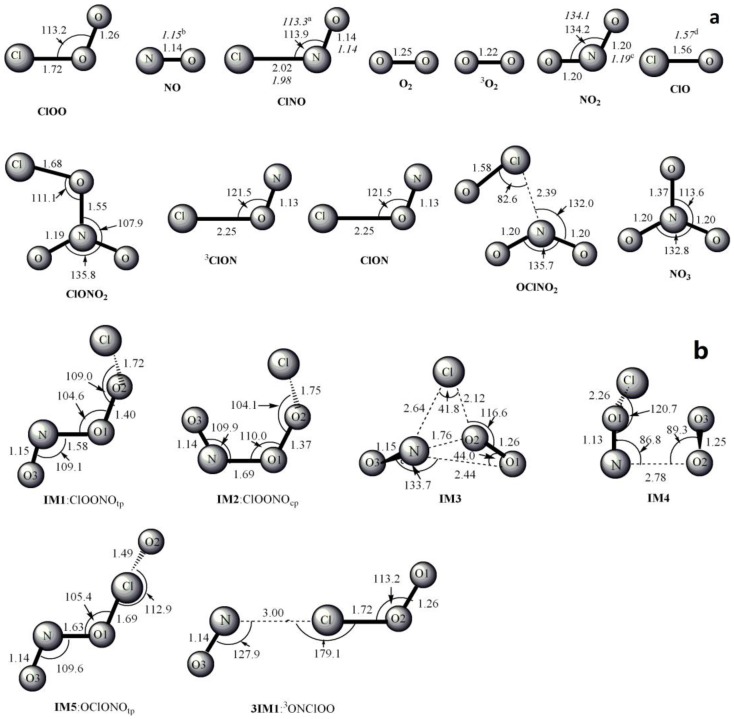

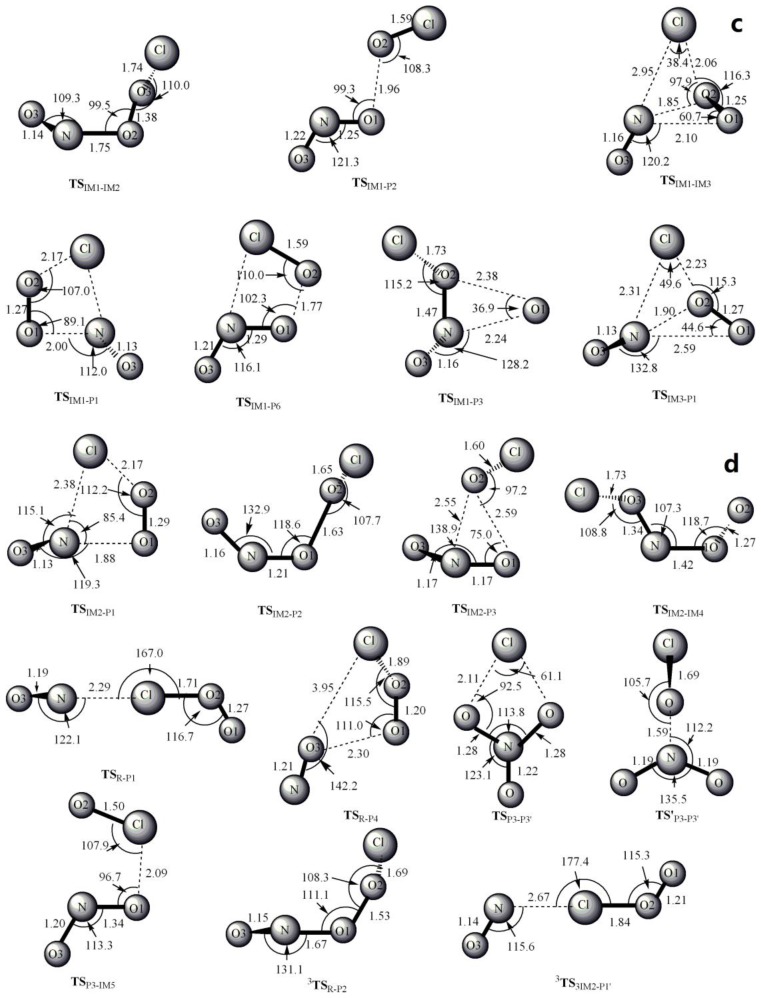
MP2/6-311 + G(2df) optimized geometries for the reactants, products, intermediates (IM) and the corresponding transition states (TS) of ClOO + NO reaction. The values in parentheses are the pertinent experimental data from the literature [48,49,50,51] and (**a**–**d**) represent refs [48,49,50,51], respectively. Bond lengths are in Å and bond angles are in degree.

**Figure 2:**
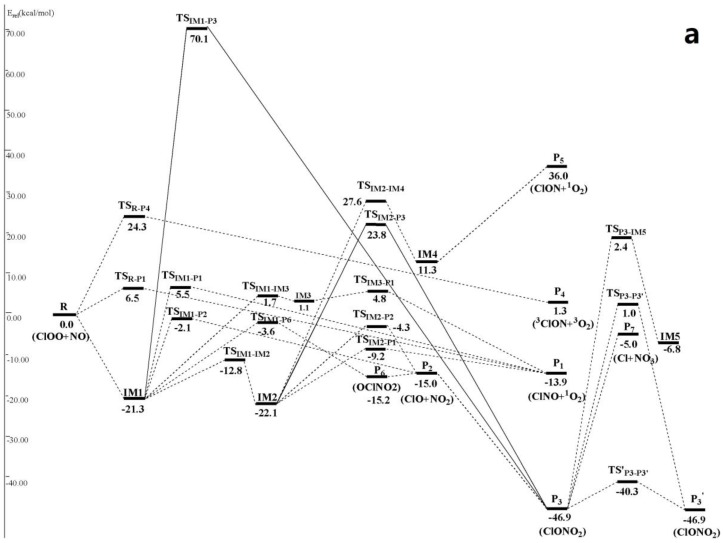

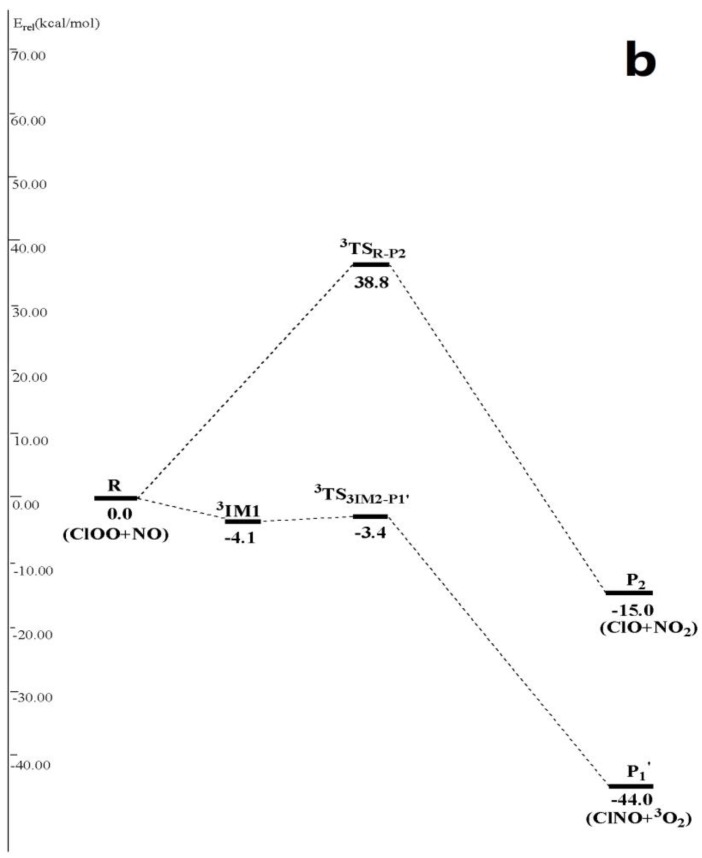
(**a**) Schematic singlet potential energy surfaces of each product channels for ClOO + NO reaction at the CCSD(T)/6-311 + G(2df)//MP2/6-311 + G(2df) + ZPE level. TS_IM1-P3_ is obtained at the CCSD (T)/6-311 + G(2df)//MP2/6-311G(d) + ZPE level, and TS_IM2-P3_ is obtained at the CCSD (T)/6-311 + G(2df)//B3LYP/6-311 + G(2df) + ZPE level; (**b**) Schematic triplet potential energy surfaces of each product channels for ClOO + NO reaction at the CCSD(T)/6-311 + G(2df)//MP2/6-311 + G(2df) + ZPE level.

The optimized geometries of the reactants, products, intermediates and transition states for ClOO + NO reaction are shown in Figure 1, respectively, along with the available experimental data from the literature. It is found that when comparison is available, the agreement between theoretical and experimental results is good, with the largest discrepancy within a factor of 0.9%. The acronyms “cp” and “tp” are used to denote the cis-perp and trans-perp forms of the isomers. The schematic profile of the PESs is depicted in Figure 2. The total energy of the reactant R (ClOO + NO) is set to be zero for reference.

#### 3.1.1. Addition Reaction Channels

It can be seen from Figure 2 that on singlet potential energy surface, the ClOO + NO reaction could be added onto O atom of ClOO• through the N-atom of NO• to form entrance intermediate with rich energies IM1 ClOONOtp (−21.3 kcal/mol). Such addition reaction will release abundant heats, making IM1 gain high chemical activity and easy to be further isomerized and dissociated. This is a typical free radical-free radical reaction mechanism. We should note that IM1 can be converted into its cis-perp conformer IM2 ClOONOcp (−22.1 kcal/mol) only by overcoming a small energy barrier 8.5 kcal/mol and passing through a transition state TS_IM1-IM2_ of –NO group rotation.

Five available dissociation and isomerization reaction channels were found from IM1 ClOONOtp (Figure 2): (1) generation channel of P_1_ ClNO + ^1^O_2_ (−13.9 kcal/mol) through a four-membered ring transition state TS_IM1-P1_ (5.5 kcal/mol) of 1, 3 Cl-atom transfer; (2) generation channel of P_2_ClO + NO_2_ (−15.0 kcal/mol) through the transition state TS_IM1-P2_ (−2.1 kcal/mol) of direct O–O bond breakage; (3) generation channel of P_6_ OClNO_2_ (−15.2 kcal/mol) through a four-membered ring transition state TS_IM1-P6_ (−3.6 kcal/mol) of synergetic O–O bond breakage and N–Cl bond formation. P_6_ could make secondary dissociation reaction and produce P_2_ through direct N–Cl bond pyrolysis; (4) generation channel of IM3 (1.1 kcal/mol) through the transition state TS_IM1-IM3_ (1.7 kcal/mol) of synergetic N–O and O–Cl bond breakages. IM3 is a complex with loose structure, which passes through the transition state TS_IM3-P1_ and produces P_1_ after overcoming a 3.7 kcal/mol energy barrier. (5) Based on structure optimization on MP2 theoretical level combined with 6-311G(d) basis set and single-point energy correction on CCSD(T)/6-311 + G(2df) level, another reaction channel could be found. Based on O–Cl transfer, IM1 passes through a three-membered ring transition state TS_IM1-P3_ (70.1 kcal/mol) and produces P_3_ ClONO_2_ (−46.9 kcal/mol). P_2_ and P_3_ could convert mutually. P_3_ could be further converted to produce IM5 OlONOtp (−6.8 kcal/mol) (through TS_P3-IM5_ (2.4 kcal/mol)), P_7_ Cl• + NO_3_ (−5.0 kcal/mol) (through generation of loose energy barrier-free variational transition state) and P_3_′ (through TS_P3-P3′_ (1.0 kcal/mol) or TS′_P3-P3′_ (−40.3 kcal/mol)). Obviously, since energies of TS_IM1-P1_, TS_IM1-IM3_ and TS_IM1-P3_ of rate-limiting step in channel (1), (4) and (5) are all positive and far higher than those of TS_IM1-P2_and TS_IM1-P6_ in channel (2) and (3), channel (1), (4) and (5) couldn’t compete with channel (2) and (3). Therefore, they are not involved in the following kinetic calculation.

Four available dissociation and isomerization reaction channels were determined from IM2 ClOONOcp (Figure 2). Firstly, IM2 could make 1,3 Cl-atom transfer through a four-membered ring transition state TS_IM2-P1_ (−9.2 kcal/mol) and produce P_1_. Secondly, IM2 also could make direct O–O bond breakage through the transition state TS_IM2-P2_ (−4.3 kcal/mol) to produce P_2_. Moreover, IM2 can be converted into isomer IM4 ClONOO (11.3 kcal/mol) through a transition state TS_IM2-IM4_ (27.6 kcal/mol) of 1,4 Cl-transfer. IM4 can be decomposed directly into P_5_ ClON + ^1^O_2_ (36.0 kcal/mol) without energy barrier. Additionally, we also discovered a reaction channel of IM2 on CCSD(T)/6-311 + G(2df)//B3LYP/6-311 + G(2df) theoretical level, which produces P3 through 1,2-OCl transfer after passing through a three-membered ring transition state TS_IM2-P3_ (23.8 kcal/mol). It is easy to know that channel (3) and (4) have to overcome big energy barriers, which are impractical in energy. As a result, they are neglected in the following kinetic calculation.

#### 3.1.2. Abstraction Reaction Channels

In Figure 2, ClOO + NO reaction has another two abstraction reaction channels on singlet and triplet potential energy surfaces. On the singlet potential energy surface, both N atom and O atom in NO• can abstract Cl atom in ClOO• directly to generate P_1_ and P_4_^3^ClON + ^3^O_2_ (1.3 kcal/mol), respectively. Their transition states are TS_R-P1_ (6.5 kcal/mol), forming TS_R-P4_ (24.3 kcal/mol). On the triplet potential energy surface, N atom in NO• can either abstract O atom in ClOO• directly and produces P_2_ through a high-energy transition state ^3^TS_R-P2_ (38.8 kcal/mol), or abstract Cl atoms and produces P_1_′ ClNO + ^3^O_2_ (−44.0 kcal/mol) after passing through a triplet van der Waals complex ^3^IM1 ^3^ONClOO (−4.1 kcal/mol) and then the transition state ^3^TS_3IM2-P1′_ (−3.4 kcal/mol). Since previous three abstraction reaction channels contain very high reaction energy barrier, the last abstraction reaction channel that produces P_1_′ is the only one feasible channel, which is considered in the following kinetic calculation.

To sum up, available channels for ClOO + NO reaction are:

R (ClOO + NO)→IM1* ClOONOtp→IM1

       →P_2_ClO + NO_2_

               →P_6_OClNO_2_→P_2_ClO + NO_2_

       →IM2 ClOONOcp→P_1_ClNO + ^1^O_2_

               →P_2_ClO + NO_2_

R (ClOO + NO)→^3^IM1* ^3^ONClOO→P_1_′ClNO + ^3^O_2_

On singlet potential energy surface, generation of IM1-2, P_6_, P_1_ and P_2_ as well as direction Cl- abstraction product P_1_′ through a triplet potential energy surface is the most feasible in view of energy (Figure 2). However, since the rate-limiting steps in their generation channels only have slight energy differences, it is difficult to determine possible reaction channels and feasible products under different temperature and pressure ranges only from the perspective of energy. Hence, the following text uses RRKM calculation to calculate rate constants and branching ratio of these competitive channels. 

### 3.2. Kinetic Calculations

**Figure 3:**
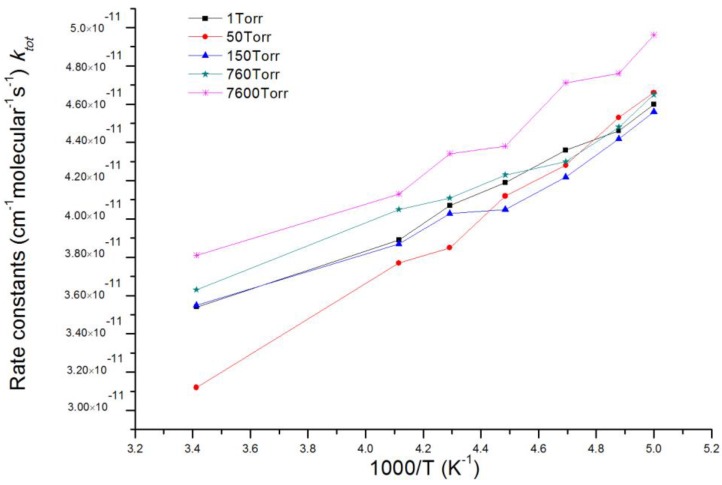
The total reaction rate constant (*k*_tot_) at various pressures of 1, 50, 150, 760 and 7600 Torr in a temperature range of 200~293 K.

Based on acquired potential energy surfaces of ClOO + NO reaction, rate constants of the overall reaction and multiple reaction channels as well as branching ratio of various products under the temperature range 200–293 K and pressure range 1~7600 Torr were calculated with MultiWell 2011 program [46,47]. Energies and molecular parameters (reaction energy barrier, rotational inertia and vibration frequency) of reactants, products, intermediates and transition states which were calculated from ab initio were used in kinetic calculation. c bottleneck of energy barrier-free reaction channels, for example, entrance channel of IM1 ClOONOtp with chemical activity, was identified using variational transition state theory (VTST) [52,53]. Therefore, we carried out restricted optimization calculation under fixed length of N-O bond in IM1 ClOONOtp and multiple reference states CASSCF(8,6)/aug-cc-pvdz. Single-point total energy usage along the reaction coordinates was corrected by using CASPT2(8,6)/aug-cc-pvdz. CASPT2//CASSCF computation was accomplished by using MOLPRO 2006 program [54,55]. Other necessary information about potential energy surfaces in the kinetic calculation were acquired on CCSD(T)/6-311 + G(2df)//MP2/6-311 + G(2df) theoretical levels. The total reaction rate constant (*k*_tot_) is the sum of rate constants of corresponding reaction channels. Table 1 shows that the RRKM theoretical values agree well with experimental values of Enami et al. [36]. within the studying temperature range. The total reaction rate constant (*k*_tot_) at various pressures of 1, 50, 150, 760 and 7600 Torr in a temperature range of 200–293 K is presented in Figure 3. It is seen that under different pressures, *k*_tot_ decreases slightly with the increase of temperature.

**Figure 4:**
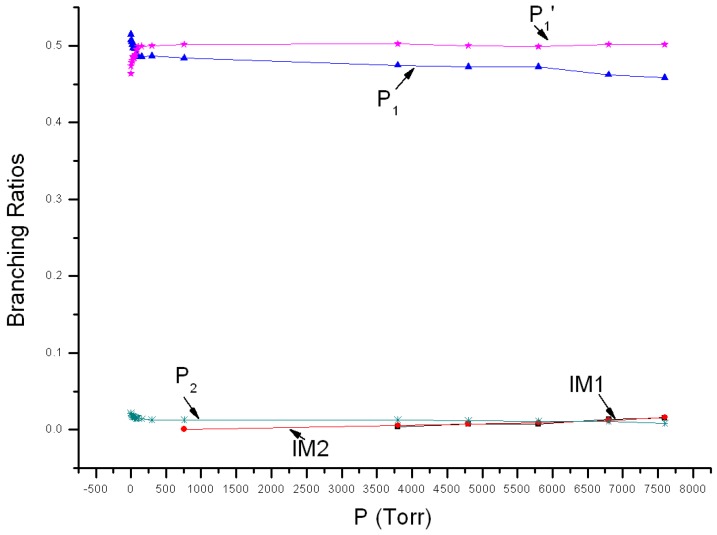
Branching ratios of various reaction channels at 213 K in a pressure range from 1 Torr to 7600 Torr.

Variations of reaction branching ratios of the overall reaction channel and reaction channels against pressure (1~7600 Torr) under 213 K are shown in Figure 4. At 213 K, P_1_ is the major product when pressure <77 Torr, showing increasing yield from 0.52 at 1 Torr to 0.49 at 77 Torr. P_1_′ is the secondary major product and its yield increases continuously from 0.46 at 1 Torr to 0.49 at 77 Torr. When pressure >77 Torr, P_1_ become the secondary major product, having 0.46 yield at 7600 Torr, while P_1_′ become the major product, having 0.50 yield at 7600 Torr. The yield of P_2_ reduces gradually with the increase of pressure. It reduces from 0.02 at 1 Torr to 0.01 at 7600 Torr. Under 213 K, the stabilization effect of intermediate could be neglected, and yields of IM1 and IM2 are smaller than 0.02. Theoretical values were compared with previous experimental data. The experiment speculated that the major product is ClNO, which echoes with the theoretical result. However, the theoretical yield of ClNO from 1~7600 Torr is about 0.98–0.96, higher than the experimental estimation (0.80). The theoretical branching ratio of secondary product NO_2_ is lower than the observed 0.18 ± 0.02. Moreover, the kinetic calculation also revealed that yields of ClOONOtp and ClOONOcp under 7600 Torr are very small and their branching ratios are about 0.02. No associated experimental data is available yet.

**Figure 5:**
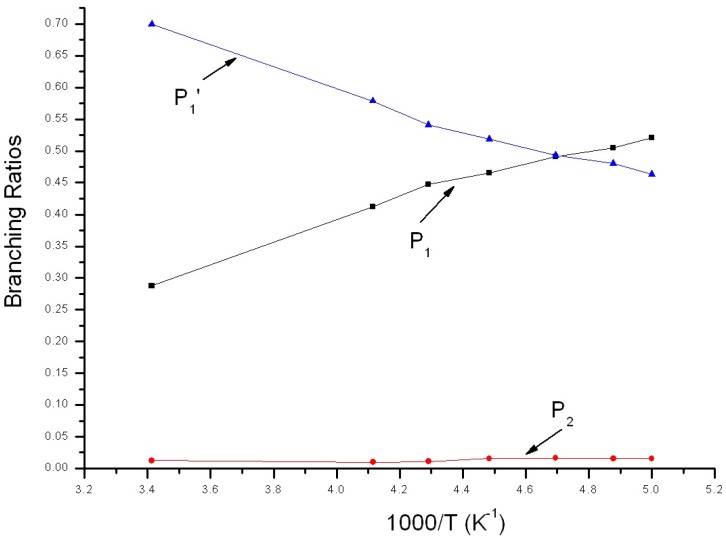
Branching ratios of various reaction channels at 1 Torr in a temperature range from 200 K to 293 K.

Reaction branching ratios of reaction channels at 1 Torr when temperature changes between 200~293 K are shown in Figure 5. Obviously, the impact stabilization effect of intermediate at 1Torr can be neglected completely. IM1 and IM2 transformed into P_1_ and P_2_ completely. In Figure 5, branching ratio of P_1_ is inversely proportional to temperature, reducing gradually from 0.52 at 200 K to 0.29 at 293 K. Branching ratio of P_2_ is also inversely proportional to temperature, reducing gradually from 0.02 at 200 K to about 0.01 at 293 K. On the contrary, branching ratio of P_1_′ is proportional to temperature, increasing continuously from 0.46 at 200 K to 0.70 at 293 K. When temperature <212 K, P_1_ is the major product and the branching ratio order of products is: P_1_ > P_1_′ > P_2_. When temperature >212 K, P_1_′ is the major product and the branching ratio order of products is: P_1_′ > P_1_ > P_2_.

**Figure 6:**
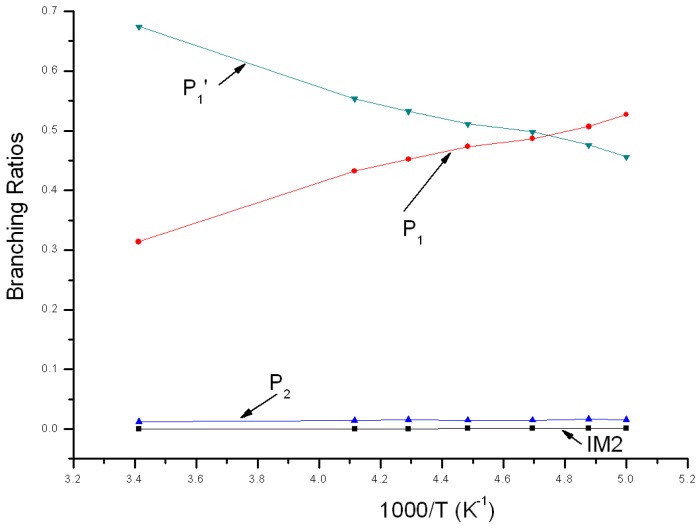
Branching ratios of various reaction channels at 760 Torr in a temperature range from 200 K to 293 K.

Variations of the reaction branching ratio of reaction channels at 760 Torr when temperature varies between 200~293 K are shown in Figure 6. When temperature >210 K, P_1_′ is the major product, with an increasing yield from 0.46 at 200 K to the peak (about 0.67) at 293 K. When temperature <210 K, P_1_ is the major product and its yield reduces gradually from 0.53 at 200 K to 0.31 at 293 K. The branching ratio of bimolecular product P_2_ declines gradually from 0.02 at 200 K to 0.01 at 293 K. The branching ratio of unimolecular product IM2 remains smaller than 0.001 within this temperature variation range, which could be neglected.

**Figure 7:**
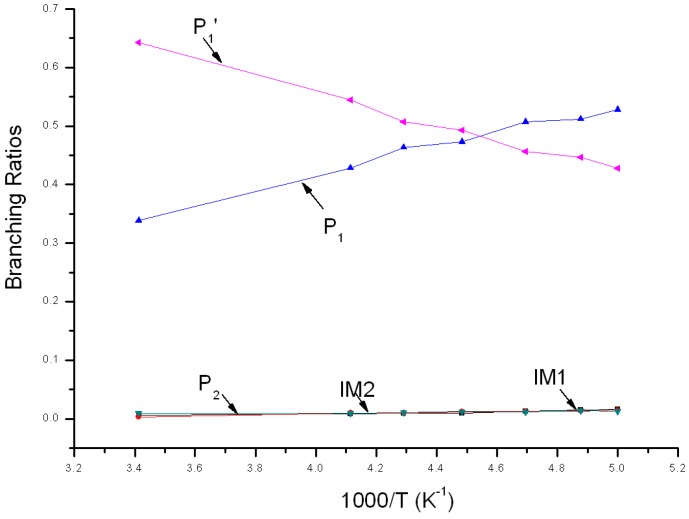
Branching ratios of various reaction channels at 7600 Torr in a temperature range from 200 K to 293 K.

Variations of the reaction branching ratio of reaction channels at 7600 Torr when temperature varies between 200~293 K are shown in Figure 7. When temperature >220 K, P_1_′ is the major product and its yield increases continuously from 0.43 at 200 K to the peak (about 0.64) at 293 K. As temperature increases from 200 K to 293 K, branching ratios of both P_1_ and P_2_ decrease, from 0.53 to 0.34 and from 0.02 to 0.01, respectively. IM1 has some yield and its branching ratio decreases from 0.02 to 0.01. The yield of IM2 ≤ 0.01 and can be neglected within this temperature range. When temperature <220 K, the branching ratio order of products is: P_1_ > P_1_′ > IM1 > P_2_ ≈ IM2. Branching ratios of products at 200 K are: 0.53 > 0.43 > 0.02 > 0.01 ≈ 0.01.

## 4. Conclusions

On singlet potential energy surface, addition-dissociation is main mechanism of ClOO + NO reaction. Firstly, N atom in NO can attack Cl atom in ClOO to generate entrance intermediate IM1ClOONOtp with rich energy, influenced by no energy barrier. IM1 can transform into its cis isomer IM2 ClOONOcp easily. Viewed from IM1 and IM2, P_1_ClNO + ^1^O_2_ is the major product and P_2_ ClO + NO_2_ is the secondary product. On triplet potential energy surface, the channel that forms loose Van Der Waals complex ^3^IM1 ^3^ONClOO and then overcome a small energy barrier to make Cl-abstraction reaction which produces P_1_′ClNO + ^3^O_2_ is the most important. The further kinetic calculation demonstrates that the total reaction rate constant (*k*_tot_) seems independent from pressure, but has a small negative temperature dependence. Within the studying pressure range, singlet biomolecular product P_1_ is the major product under low temperature, and triplet product fragment P_1_′ is the major product under high temperature. Monomolecular products IM1 and IM2 only have very small yields under low temperature and high pressure. The stabilization effect of intermediate at 213 K could be neglected. The yields of IM1 and IM2 remain smaller than 0.02. Within the studying pressure range, ClNO is the major product, which agrees with the experimental expectation.

## Figures and Tables

**Table 1:** At 150 Torr, experimental data and the theoretical calculation results of the total reaction rate constant (*k*_tot_).

T(K)/*k*_tot_ (×10^−11^ cm^3^ mol^−1^ s^−1^)	205	213	223	233	243
Experimental data ^a^	4.3 ± 0.8	4.5 ± 0.9	4.2 ± 0.8	4.9 ± 1.0	5.5 ± 1.2
Theoretical calculation results (RRKM)	4.4	4.2	4.1	4.0	3.9

^a^ represent refs. [36].
